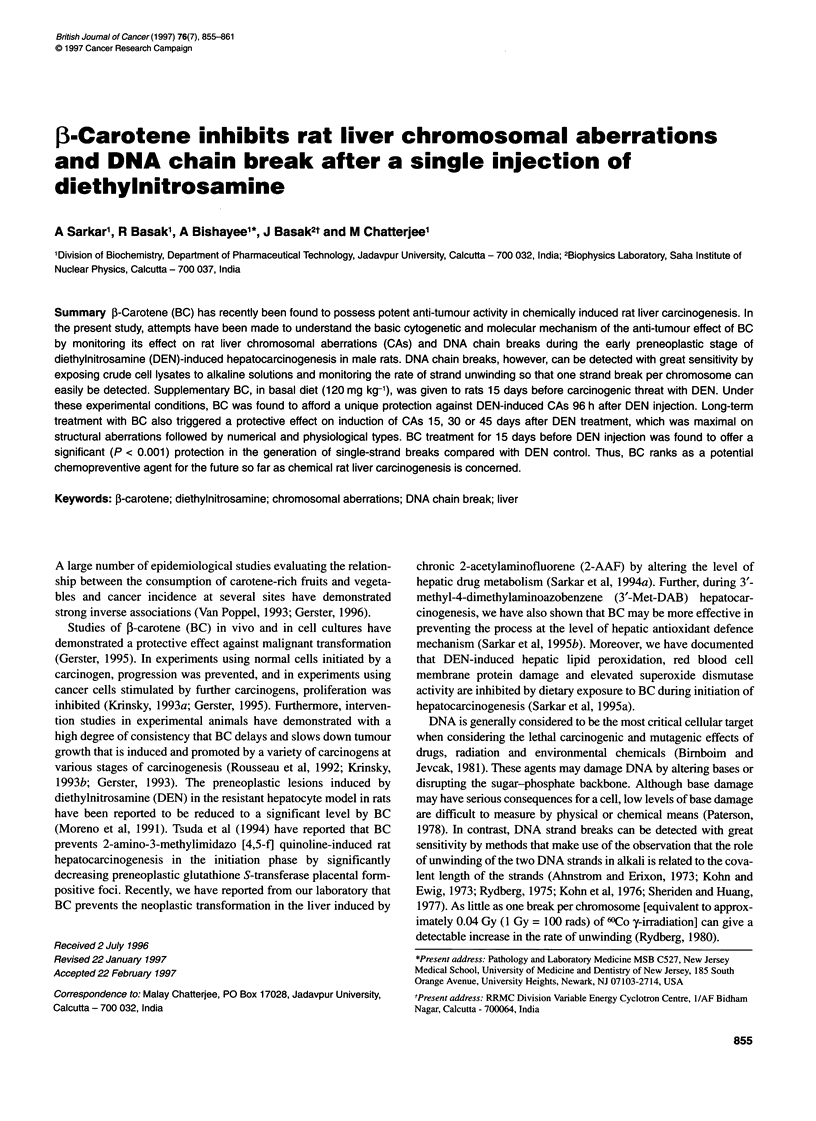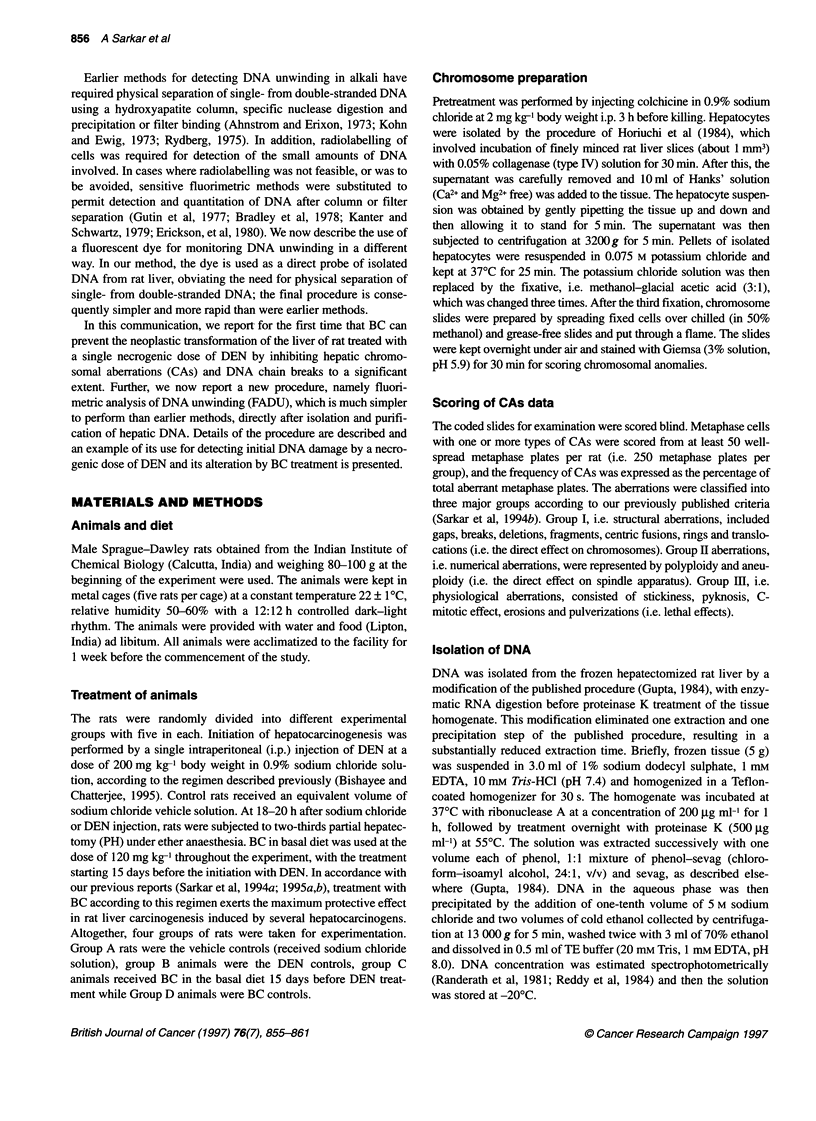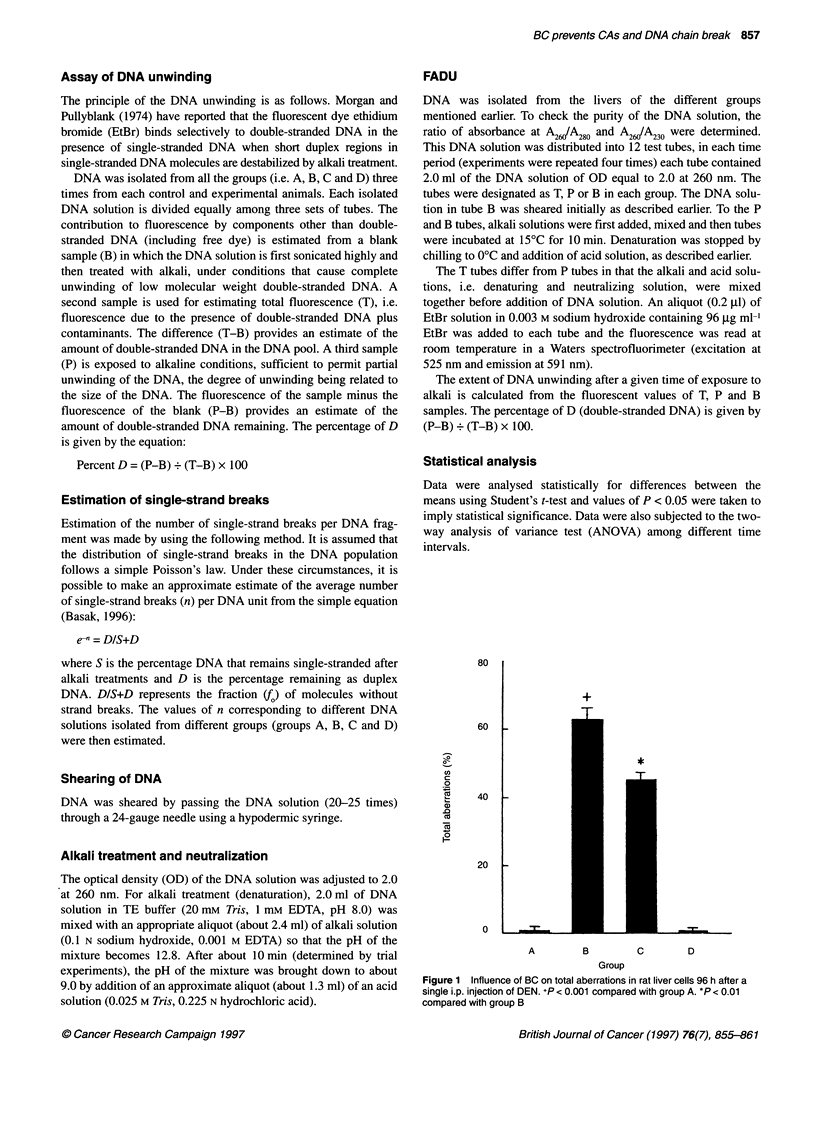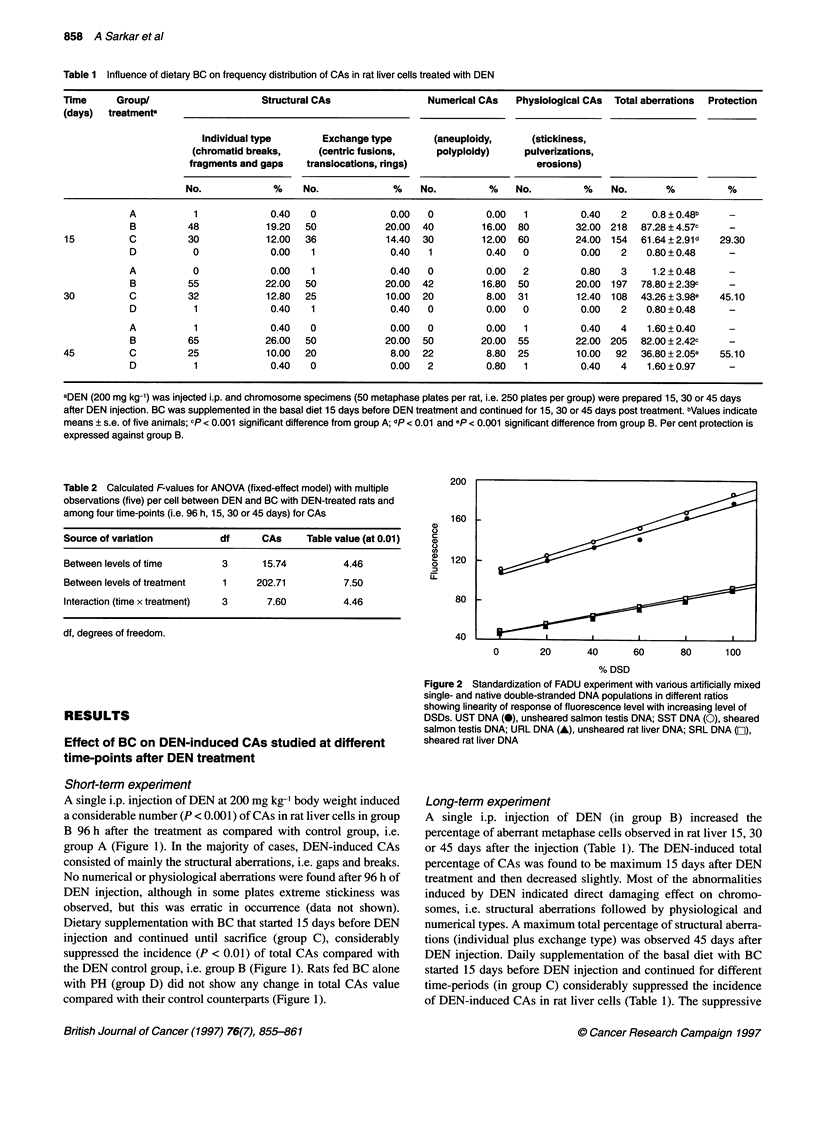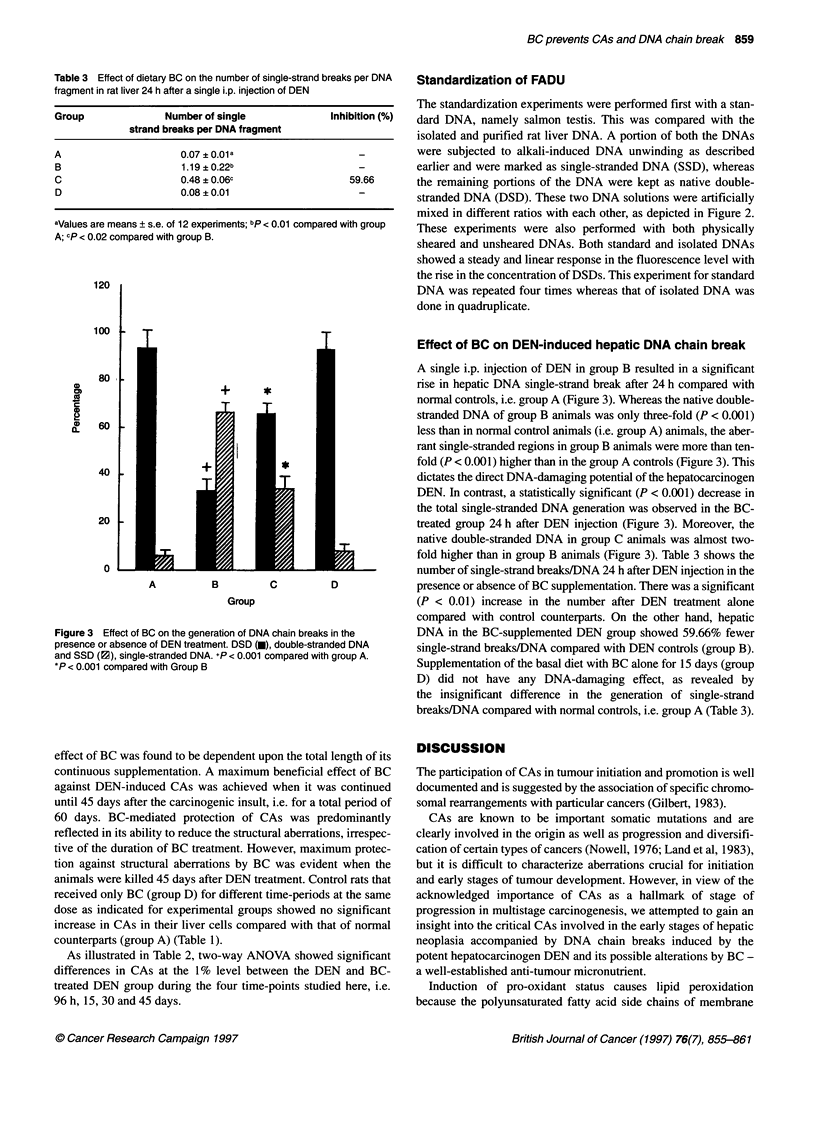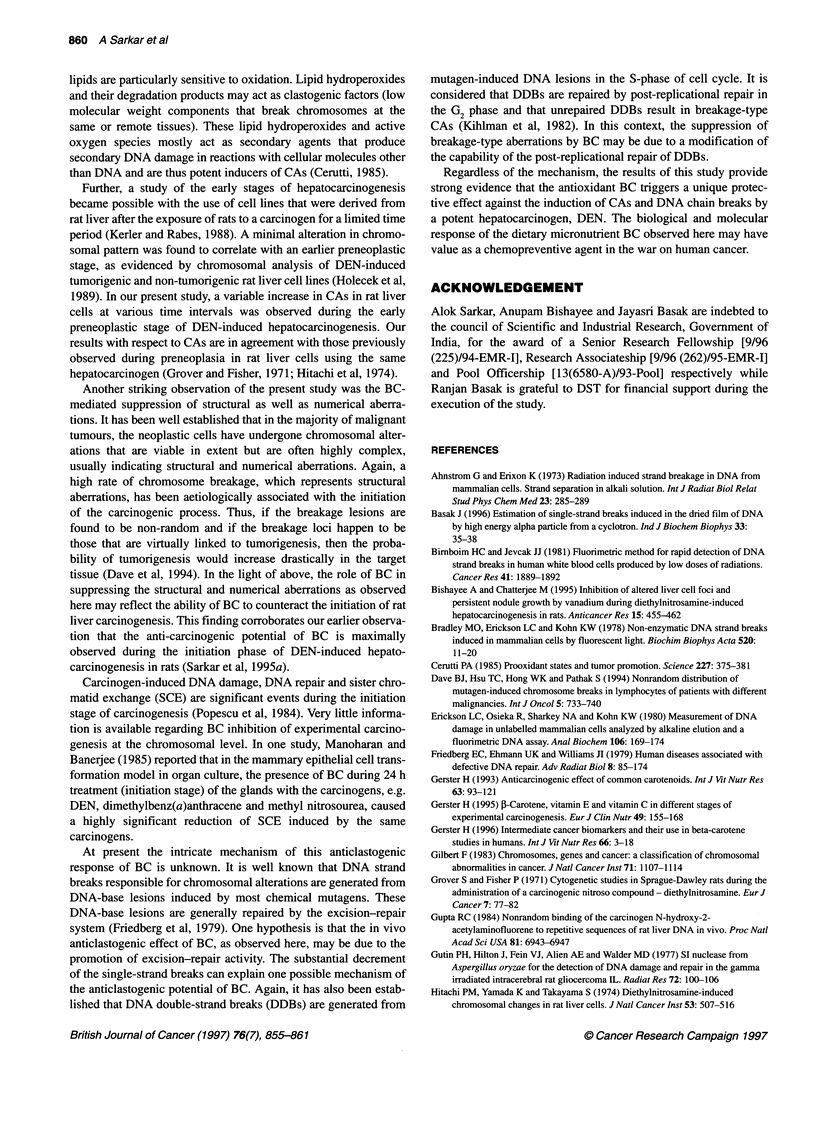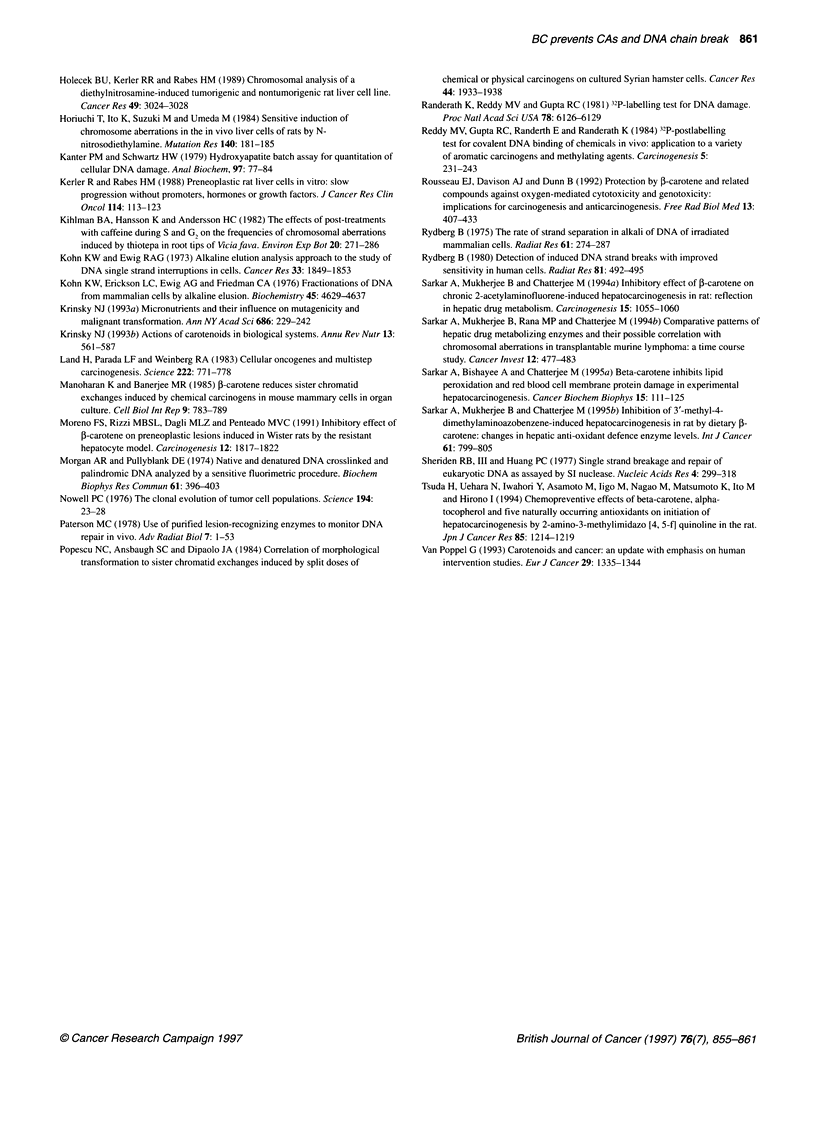# Beta-carotene inhibits rat liver chromosomal aberrations and DNA chain break after a single injection of diethylnitrosamine.

**DOI:** 10.1038/bjc.1997.475

**Published:** 1997

**Authors:** A. Sarkar, R. Basak, A. Bishayee, J. Basak, M. Chatterjee

**Affiliations:** Department of Pharmaceutical Technology, Jadavpur University, Calcutta, India.

## Abstract

Beta-carotene (BC) has recently been found to possess potent anti-tumour activity in chemically induced rat liver carcinogenesis. In the present study, attempts have been made to understand the basic cytogenetic and molecular mechanism of the anti-tumour effect of BC by monitoring its effect on rat liver chromosomal aberrations (CAs) and DNA chain breaks during the early preneoplastic stage of diethylnitrosamine (DEN)-induced hepatocarcinogenesis in male rats. DNA chain breaks, however, can be detected with great sensitivity by exposing crude cell lysates to alkaline solutions and monitoring the rate of strand unwinding so that one strand break per chromosome can easily be detected. Supplementary BC, in basal diet (120 mg kg[-1]), was given to rats 15 days before carcinogenic threat with DEN. Under these experimental conditions, BC was found to afford a unique protection against DEN-induced CAs 96 h after DEN injection. Long-term treatment with BC also triggered a protective effect on induction of CAs 15, 30 or 45 days after DEN treatment, which was maximal on structural aberrations followed by numerical and physiological types. BC treatment for 15 days before DEN injection was found to offer a significant (P < 0.001) protection in the generation of single-strand breaks compared with DEN control. Thus, BC ranks as a potential chemopreventive agent for the future so far as chemical rat liver carcinogenesis is concerned.


British Journal of Cancer (1997) 76(7), 855–861
© 1997 Cancer Research Campaign

### β-Carotene inhibits rat liver chromosomal aberrations and DNA chain break after a single injection of diethylnitrosamine

**A Sarkar[1], R Basak[1], A Bishayee[1*], J Basak[2†] and M Chatterjee[1]**

[1]Division of Biochemistry, Department of Pharmaceutical Technology, Jadavpur University, Calcutta – 700 032, India; [2]Biophysics Laboratory, Saha Institute of Nuclear Physics, Calcutta – 700 037, India

**Summary** β-Carotene (BC) has recently been found to possess potent anti-tumour activity in chemically induced rat liver carcinogenesis. In the present study, attempts have been made to understand the basic cytogenetic and molecular mechanism of the anti-tumour effect of BC by monitoring its effect on rat liver chromosomal aberrations (CAs) and DNA chain breaks during the early preneoplastic stage of diethylnitrosamine (DEN)-induced hepatocarcinogenesis in male rats. DNA chain breaks, however, can be detected with great sensitivity by exposing crude cell lysates to alkaline solutions and monitoring the rate of strand unwinding so that one strand break per chromosome can easily be detected. Supplementary BC, in basal diet (120 mg $kg^{-1}$), was given to rats 15 days before carcinogenic threat with DEN. Under these experimental conditions, BC was found to afford a unique protection against DEN-induced CAs 96 h after DEN injection. Long-term treatment with BC also triggered a protective effect on induction of CAs 15, 30 or 45 days after DEN treatment, which was maximal on structural aberrations followed by numerical and physiological types. BC treatment for 15 days before DEN injection was found to offer a significant ($P < 0.001$) protection in the generation of single-strand breaks compared with DEN control. Thus, BC ranks as a potential chemopreventive agent for the future so far as chemical rat liver carcinogenesis is concerned.

**Keywords:** β-carotene; diethylnitrosamine; chromosomal aberrations; DNA chain break; liver

A large number of epidemiological studies evaluating the relationship between the consumption of carotene-rich fruits and vegetables and cancer incidence at several sites have demonstrated strong inverse associations (Van Poppel, 1993; Gerster, 1996).

Studies of β-carotene (BC) in vivo and in cell cultures have demonstrated a protective effect against malignant transformation (Gerster, 1995). In experiments using normal cells initiated by a carcinogen, progression was prevented, and in experiments using cancer cells stimulated by further carcinogens, proliferation was inhibited (Krinsky, 1993*a*; Gerster, 1995). Furthermore, intervention studies in experimental animals have demonstrated with a high degree of consistency that BC delays and slows down tumour growth that is induced and promoted by a variety of carcinogens at various stages of carcinogenesis (Rousseau et al, 1992; Krinsky, 1993*b*; Gerster, 1993). The preneoplastic lesions induced by diethylnitrosamine (DEN) in the resistant hepatocyte model in rats have been reported to be reduced to a significant level by BC (Moreno et al, 1991). Tsuda et al (1994) have reported that BC prevents 2-amino-3-methylimidazo [4,5-f] quinoline-induced rat hepatocarcinogenesis in the initiation phase by significantly decreasing preneoplastic glutathione *S*-transferase placental form-positive foci. Recently, we have reported from our laboratory that BC prevents the neoplastic transformation in the liver induced by chronic 2-acetylaminofluorene (2-AAF) by altering the level of hepatic drug metabolism (Sarkar et al, 1994*a*). Further, during 3′-methyl-4-dimethylaminoazobenzene (3′-Met-DAB) hepatocarcinogenesis, we have also shown that BC may be more effective in preventing the process at the level of hepatic antioxidant defence mechanism (Sarkar et al, 1995*b*). Moreover, we have documented that DEN-induced hepatic lipid peroxidation, red blood cell membrane protein damage and elevated superoxide dismutase activity are inhibited by dietary exposure to BC during initiation of hepatocarcinogenesis (Sarkar et al, 1995a).

DNA is generally considered to be the most critical cellular target when considering the lethal carcinogenic and mutagenic effects of drugs, radiation and environmental chemicals (Birnboim and Jevcak, 1981). These agents may damage DNA by altering bases or disrupting the sugar–phosphate backbone. Although base damage may have serious consequences for a cell, low levels of base damage are difficult to measure by physical or chemical means (Paterson, 1978). In contrast, DNA strand breaks can be detected with great sensitivity by methods that make use of the observation that the role of unwinding of the two DNA strands in alkali is related to the covalent length of the strands (Ahnstrom and Erixon, 1973; Kohn and Ewig, 1973; Rydberg, 1975; Kohn et al, 1976; Sheriden and Huang, 1977). As little as one break per chromosome [equivalent to approximately 0.04 Gy (1 Gy = 100 rads) of $^{60}$Co γ-irradiation] can give a detectable increase in the rate of unwinding (Rydberg, 1980).

*Received 2 July 1996*
*Revised 22 January 1997*
*Accepted 22 February 1997*

*Correspondence to:* Malay Chatterjee, PO Box 17028, Jadavpur University, Calcutta – 700 032, India

**Present address:* Pathology and Laboratory Medicine MSB C527, New Jersey Medical School, University of Medicine and Dentistry of New Jersey, 185 South Orange Avenue, University Heights, Newark, NJ 07103-2714, USA

†*Present address:* RRMC Division Variable Energy Cyclotron Centre, 1/AF Bidham Nagar, Calcutta - 700064, India

Earlier methods for detecting DNA unwinding in alkali have required physical separation of single- from double-stranded DNA using a hydroxyapatite column, specific nuclease digestion and precipitation or filter binding (Ahnstrom and Erixon, 1973; Kohn and Ewig, 1973; Rydberg, 1975). In addition, radiolabelling of cells was required for detection of the small amounts of DNA involved. In cases where radiolabelling was not feasible, or was to be avoided, sensitive fluorimetric methods were substituted to permit detection and quantitation of DNA after column or filter separation (Gutin et al, 1977; Bradley et al, 1978; Kanter and Schwartz, 1979; Erickson, et al, 1980). We now describe the use of a fluorescent dye for monitoring DNA unwinding in a different way. In our method, the dye is used as a direct probe of isolated DNA from rat liver, obviating the need for physical separation of single- from double-stranded DNA; the final procedure is consequently simpler and more rapid than were earlier methods.

In this communication, we report for the first time that BC can prevent the neoplastic transformation of the liver of rat treated with a single necrogenic dose of DEN by inhibiting hepatic chromosomal aberrations (CAs) and DNA chain breaks to a significant extent. Further, we now report a new procedure, namely fluorimetric analysis of DNA unwinding (FADU), which is much simpler to perform than earlier methods, directly after isolation and purification of hepatic DNA. Details of the procedure are described and an example of its use for detecting initial DNA damage by a necrogenic dose of DEN and its alteration by BC treatment is presented.

### MATERIALS AND METHODS

### Animals and diet

Male Sprague–Dawley rats obtained from the Indian Institute of Chemical Biology (Calcutta, India) and weighing 80–100 g at the beginning of the experiment were used. The animals were kept in metal cages (five rats per cage) at a constant temperature 22 ± 1°C, relative humidity 50–60% with a 12:12 h controlled dark–light rhythm. The animals were provided with water and food (Lipton, India) ad libitum. All animals were acclimatized to the facility for 1 week before the commencement of the study.

### Treatment of animals

The rats were randomly divided into different experimental groups with five in each. Initiation of hepatocarcinogenesis was performed by a single intraperitoneal (i.p.) injection of DEN at a dose of 200 mg $kg^{-1}$ body weight in 0.9% sodium chloride solution, according to the regimen described previously (Bishayee and Chatterjee, 1995). Control rats received an equivalent volume of sodium chloride vehicle solution. At 18–20 h after sodium chloride or DEN injection, rats were subjected to two-thirds partial hepatectomy (PH) under ether anaesthesia. BC in basal diet was used at the dose of 120 mg $kg^{-1}$ throughout the experiment, with the treatment starting 15 days before the initiation with DEN. In accordance with our previous reports (Sarkar et al, 1994*a*; 1995*a*,*b*), treatment with BC according to this regimen exerts the maximum protective effect in rat liver carcinogenesis induced by several hepatocarcinogens. Altogether, four groups of rats were taken for experimentation. Group A rats were the vehicle controls (received sodium chloride solution), group B animals were the DEN controls, group C animals received BC in the basal diet 15 days before DEN treatment while Group D animals were BC controls.

### Chromosome preparation

Pretreatment was performed by injecting colchicine in 0.9% sodium chloride at 2 mg $kg^{-1}$ body weight i.p. 3 h before killing. Hepatocytes were isolated by the procedure of Horiuchi et al (1984), which involved incubation of finely minced rat liver slices (about 1 $mm^3$) with 0.05% collagenase (type IV) solution for 30 min. After this, the supernatant was carefully removed and 10 ml of Hanks' solution ($Ca^{2+}$ and $Mg^{2+}$ free) was added to the tissue. The hepatocyte suspension was obtained by gently pipetting the tissue up and down and then allowing it to stand for 5 min. The supernatant was then subjected to centrifugation at 3200 ***g*** for 5 min. Pellets of isolated hepatocytes were resuspended in 0.075 M potassium chloride and kept at 37°C for 25 min. The potassium chloride solution was then replaced by the fixative, i.e. methanol–glacial acetic acid (3:1), which was changed three times. After the third fixation, chromosome slides were prepared by spreading fixed cells over chilled (in 50% methanol) and grease-free slides and put through a flame. The slides were kept overnight under air and stained with Giemsa (3% solution, pH 5.9) for 30 min for scoring chromosomal anomalies.

### Scoring of CAs data

The coded slides for examination were scored blind. Metaphase cells with one or more types of CAs were scored from at least 50 well-spread metaphase plates per rat (i.e. 250 metaphase plates per group), and the frequency of CAs was expressed as the percentage of total aberrant metaphase plates. The aberrations were classified into three major groups according to our previously published criteria (Sarkar et al, 1994*b*). Group I, i.e. structural aberrations, included gaps, breaks, deletions, fragments, centric fusions, rings and translocations (i.e. the direct effect on chromosomes). Group II aberrations, i.e. numerical aberrations, were represented by polyploidy and aneuploidy (i.e. the direct effect on spindle apparatus). Group III, i.e. physiological aberrations, consisted of stickiness, pyknosis, C-mitotic effect, erosions and pulverizations (i.e. lethal effects).

### Isolation of DNA

DNA was isolated from the frozen hepatectomized rat liver by a modification of the published procedure (Gupta, 1984), with enzymatic RNA digestion before proteinase K treatment of the tissue homogenate. This modification eliminated one extraction and one precipitation step of the published procedure, resulting in a substantially reduced extraction time. Briefly, frozen tissue (5 g) was suspended in 3.0 ml of 1% sodium dodecyl sulphate, 1 mM EDTA, 10 mM *Tris*-HCl (pH 7.4) and homogenized in a Teflon-coated homogenizer for 30 s. The homogenate was incubated at 37°C with ribonuclease A at a concentration of 200 μg $ml^{-1}$ for 1 h, followed by treatment overnight with proteinase K (500 μg $ml^{-1}$) at 55°C. The solution was extracted successively with one volume each of phenol, 1:1 mixture of phenol–sevag (chloroform–isoamyl alcohol, 24:1, v/v) and sevag, as described elsewhere (Gupta, 1984). DNA in the aqueous phase was then precipitated by the addition of one-tenth volume of 5 M sodium chloride and two volumes of cold ethanol collected by centrifugation at 13 000 ***g*** for 5 min, washed twice with 3 ml of 70% ethanol and dissolved in 0.5 ml of TE buffer (20 mM Tris, 1 mM EDTA, pH 8.0). DNA concentration was estimated spectrophotometrically (Randerath et al, 1981; Reddy et al, 1984) and then the solution was stored at –20°C.

© Cancer Research Campaign 1997

### Assay of DNA unwinding

The principle of the DNA unwinding is as follows. Morgan and Pullyblank (1974) have reported that the fluorescent dye ethidium bromide (EtBr) binds selectively to double-stranded DNA in the presence of single-stranded DNA when short duplex regions in single-stranded DNA molecules are destabilized by alkali treatment.

DNA was isolated from all the groups (i.e. A, B, C and D) three times from each control and experimental animals. Each isolated DNA solution is divided equally among three sets of tubes. The contribution to fluorescence by components other than double-stranded DNA (including free dye) is estimated from a blank sample (B) in which the DNA solution is first sonicated highly and then treated with alkali, under conditions that cause complete unwinding of low molecular weight double-stranded DNA. A second sample is used for estimating total fluorescence (T), i.e. fluorescence due to the presence of double-stranded DNA plus contaminants. The difference (T–B) provides an estimate of the amount of double-stranded DNA in the DNA pool. A third sample (P) is exposed to alkaline conditions, sufficient to permit partial unwinding of the DNA, the degree of unwinding being related to the size of the DNA. The fluorescence of the sample minus the fluorescence of the blank (P–B) provides an estimate of the amount of double-stranded DNA remaining. The percentage of *D* is given by the equation:

$$\text{Percent } D = (\text{P–B}) \div (\text{T–B}) \times 100$$

### Estimation of single-strand breaks

Estimation of the number of single-strand breaks per DNA fragment was made by using the following method. It is assumed that the distribution of single-strand breaks in the DNA population follows a simple Poisson's law. Under these circumstances, it is possible to make an approximate estimate of the average number of single-strand breaks ($n$) per DNA unit from the simple equation (Basak, 1996):

$$e^{-n} = D/S{+}D$$

where $S$ is the percentage DNA that remains single-stranded after alkali treatments and $D$ is the percentage remaining as duplex DNA. $D/S{+}D$ represents the fraction ($f_0$) of molecules without strand breaks. The values of $n$ corresponding to different DNA solutions isolated from different groups (groups A, B, C and D) were then estimated.

### Shearing of DNA

DNA was sheared by passing the DNA solution (20–25 times) through a 24-gauge needle using a hypodermic syringe.

### Alkali treatment and neutralization

The optical density (OD) of the DNA solution was adjusted to 2.0 at 260 nm. For alkali treatment (denaturation), 2.0 ml of DNA solution in TE buffer (20 mM *Tris*, 1 mM EDTA, pH 8.0) was mixed with an appropriate aliquot (about 2.4 ml) of alkali solution (0.1 N sodium hydroxide, 0.001 M EDTA) so that the pH of the mixture becomes 12.8. After about 10 min (determined by trial experiments), the pH of the mixture was brought down to about 9.0 by addition of an approximate aliquot (about 1.3 ml) of an acid solution (0.025 M *Tris*, 0.225 N hydrochloric acid).

### FADU

DNA was isolated from the livers of the different groups mentioned earlier. To check the purity of the DNA solution, the ratio of absorbance at $A_{260}/A_{280}$ and $A_{260}/A_{230}$ were determined. This DNA solution was distributed into 12 test tubes, in each time period (experiments were repeated four times) each tube contained 2.0 ml of the DNA solution of OD equal to 2.0 at 260 nm. The tubes were designated as T, P or B in each group. The DNA solution in tube B was sheared initially as described earlier. To the P and B tubes, alkali solutions were first added, mixed and then tubes were incubated at 15°C for 10 min. Denaturation was stopped by chilling to 0°C and addition of acid solution, as described earlier.

The T tubes differ from P tubes in that the alkali and acid solutions, i.e. denaturing and neutralizing solution, were mixed together before addition of DNA solution. An aliquot (0.2 µl) of EtBr solution in 0.003 M sodium hydroxide containing 96 µg $ml^{-1}$ EtBr was added to each tube and the fluorescence was read at room temperature in a Waters spectrofluorimeter (excitation at 525 nm and emission at 591 nm).

The extent of DNA unwinding after a given time of exposure to alkali is calculated from the fluorescent values of T, P and B samples. The percentage of D (double-stranded DNA) is given by (P–B) ÷ (T–B) × 100.

### Statistical analysis

Data were analysed statistically for differences between the means using Student's *t*-test and values of $P < 0.05$ were taken to imply statistical significance. Data were also subjected to the two-way analysis of variance test (ANOVA) among different time intervals.

**Figure 1** Influence of BC on total aberrations in rat liver cells 96 h after a single i.p. injection of DEN. $^{+}P < 0.001$ compared with group A. $^{*}P < 0.01$ compared with group B

© Cancer Research Campaign 1997 

**Table 1** Influence of dietary BC on frequency distribution of CAs in rat liver cells treated with DEN

| Time (days) | Group/ treatment[a] | Structural CAs: Individual type (chromatid breaks, fragments and gaps | | Structural CAs: Exchange type (centric fusions, translocations, rings) | | Numerical CAs (aneuploidy, polyploidy) | | Physiological CAs (stickiness, pulverizations, erosions) | | Total aberrations | | Protection |
|---|---|---|---|---|---|---|---|---|---|---|---|---|
| | | No. | % | No. | % | No. | % | No. | % | No. | % | % |
| | A | 1 | 0.40 | 0 | 0.00 | 0 | 0.00 | 1 | 0.40 | 2 | 0.8 ± 0.48[b] | – |
| | B | 48 | 19.20 | 50 | 20.00 | 40 | 16.00 | 80 | 32.00 | 218 | 87.28 ± 4.57[c] | – |
| 15 | C | 30 | 12.00 | 36 | 14.40 | 30 | 12.00 | 60 | 24.00 | 154 | 61.64 ± 2.91[d] | 29.30 |
| | D | 0 | 0.00 | 1 | 0.40 | 1 | 0.40 | 0 | 0.00 | 2 | 0.80 ± 0.48 | – |
| | A | 0 | 0.00 | 1 | 0.40 | 0 | 0.00 | 2 | 0.80 | 3 | 1.2 ± 0.48 | – |
| | B | 55 | 22.00 | 50 | 20.00 | 42 | 16.80 | 50 | 20.00 | 197 | 78.80 ± 2.39[c] | – |
| 30 | C | 32 | 12.80 | 25 | 10.00 | 20 | 8.00 | 31 | 12.40 | 108 | 43.26 ± 3.98[e] | 45.10 |
| | D | 1 | 0.40 | 1 | 0.40 | 0 | 0.00 | 0 | 0.00 | 2 | 0.80 ± 0.48 | – |
| | A | 1 | 0.40 | 0 | 0.00 | 0 | 0.00 | 1 | 0.40 | 4 | 1.60 ± 0.40 | – |
| | B | 65 | 26.00 | 50 | 20.00 | 50 | 20.00 | 55 | 22.00 | 205 | 82.00 ± 2.42[c] | – |
| 45 | C | 25 | 10.00 | 20 | 8.00 | 22 | 8.80 | 25 | 10.00 | 92 | 36.80 ± 2.05[e] | 55.10 |
| | D | 1 | 0.40 | 0 | 0.00 | 2 | 0.80 | 1 | 0.40 | 4 | 1.60 ± 0.97 | – |

[a]DEN (200 mg $kg^{-1}$) was injected i.p. and chromosome specimens (50 metaphase plates per rat, i.e. 250 plates per group) were prepared 15, 30 or 45 days after DEN injection. BC was supplemented in the basal diet 15 days before DEN treatment and continued for 15, 30 or 45 days post treatment. [b]Values indicate means ± s.e. of five animals; [c]$P < 0.001$ significant difference from group A; [d]$P < 0.01$ and [e]$P < 0.001$ significant difference from group B. Per cent protection is expressed against group B.

**Table 2** Calculated *F*-values for ANOVA (fixed-effect model) with multiple observations (five) per cell between DEN and BC with DEN-treated rats and among four time-points (i.e. 96 h, 15, 30 or 45 days) for CAs

| Source of variation | df | CAs | Table value (at 0.01) |
|---|---|---|---|
| Between levels of time | 3 | 15.74 | 4.46 |
| Between levels of treatment | 1 | 202.71 | 7.50 |
| Interaction (time × treatment) | 3 | 7.60 | 4.46 |

df, degrees of freedom.

**Figure 2** Standardization of FADU experiment with various artificially mixed single- and native double-stranded DNA populations in different ratios showing linearity of response of fluorescence level with increasing level of DSDs. UST DNA (●), unsheared salmon testis DNA; SST DNA (○), sheared salmon testis DNA; URL DNA (▲), unsheared rat liver DNA; SRL DNA (□), sheared rat liver DNA

### RESULTS

### Effect of BC on DEN-induced CAs studied at different time-points after DEN treatment

*Short-term experiment*

A single i.p. injection of DEN at 200 mg $kg^{-1}$ body weight induced a considerable number ($P < 0.001$) of CAs in rat liver cells in group B 96 h after the treatment as compared with control group, i.e. group A (Figure 1). In the majority of cases, DEN-induced CAs consisted of mainly the structural aberrations, i.e. gaps and breaks. No numerical or physiological aberrations were found after 96 h of DEN injection, although in some plates extreme stickiness was observed, but this was erratic in occurrence (data not shown). Dietary supplementation with BC that started 15 days before DEN injection and continued until sacrifice (group C), considerably suppressed the incidence ($P < 0.01$) of total CAs compared with the DEN control group, i.e. group B (Figure 1). Rats fed BC alone with PH (group D) did not show any change in total CAs value compared with their control counterparts (Figure 1).

*Long-term experiment*

A single i.p. injection of DEN (in group B) increased the percentage of aberrant metaphase cells observed in rat liver 15, 30 or 45 days after the injection (Table 1). The DEN-induced total percentage of CAs was found to be maximum 15 days after DEN treatment and then decreased slightly. Most of the abnormalities induced by DEN indicated direct damaging effect on chromosomes, i.e. structural aberrations followed by physiological and numerical types. A maximum total percentage of structural aberrations (individual plus exchange type) was observed 45 days after DEN injection. Daily supplementation of the basal diet with BC started 15 days before DEN injection and continued for different time-periods (in group C) considerably suppressed the incidence of DEN-induced CAs in rat liver cells (Table 1). The suppressive

© Cancer Research Campaign 1997

Table 3 Effect of dietary BC on the number of single-strand breaks per DNA fragment in rat liver 24 h after a single i.p. injection of DEN

| Group | Number of single strand breaks per DNA fragment | Inhibition (%) |
|---|---|---|
| A | 0.07 ± 0.01[a] | – |
| B | 1.19 ± 0.22[b] | – |
| C | 0.48 ± 0.06[c] | 59.66 |
| D | 0.08 ± 0.01 | – |

[a]Values are means ± s.e. of 12 experiments; [b]$P < 0.01$ compared with group A; [c]$P < 0.02$ compared with group B.

**Figure 3** Effect of BC on the generation of DNA chain breaks in the presence or absence of DEN treatment. DSD (■), double-stranded DNA and SSD (▨), single-stranded DNA. +$P < 0.001$ compared with group A. *$P < 0.001$ compared with Group B

effect of BC was found to be dependent upon the total length of its continuous supplementation. A maximum beneficial effect of BC against DEN-induced CAs was achieved when it was continued until 45 days after the carcinogenic insult, i.e. for a total period of 60 days. BC-mediated protection of CAs was predominantly reflected in its ability to reduce the structural aberrations, irrespective of the duration of BC treatment. However, maximum protection against structural aberrations by BC was evident when the animals were killed 45 days after DEN treatment. Control rats that received only BC (group D) for different time-periods at the same dose as indicated for experimental groups showed no significant increase in CAs in their liver cells compared with that of normal counterparts (group A) (Table 1).

As illustrated in Table 2, two-way ANOVA showed significant differences in CAs at the 1% level between the DEN and BC-treated DEN group during the four time-points studied here, i.e. 96 h, 15, 30 and 45 days.

### Standardization of FADU

The standardization experiments were performed first with a standard DNA, namely salmon testis. This was compared with the isolated and purified rat liver DNA. A portion of both the DNAs were subjected to alkali-induced DNA unwinding as described earlier and were marked as single-stranded DNA (SSD), whereas the remaining portions of the DNA were kept as native double-stranded DNA (DSD). These two DNA solutions were artificially mixed in different ratios with each other, as depicted in Figure 2. These experiments were also performed with both physically sheared and unsheared DNAs. Both standard and isolated DNAs showed a steady and linear response in the fluorescence level with the rise in the concentration of DSDs. This experiment for standard DNA was repeated four times whereas that of isolated DNA was done in quadruplicate.

### Effect of BC on DEN-induced hepatic DNA chain break

A single i.p. injection of DEN in group B resulted in a significant rise in hepatic DNA single-strand break after 24 h compared with normal controls, i.e. group A (Figure 3). Whereas the native double-stranded DNA of group B animals was only three-fold ($P < 0.001$) less than in normal control animals (i.e. group A) animals, the aberrant single-stranded regions in group B animals were more than ten-fold ($P < 0.001$) higher than in the group A controls (Figure 3). This dictates the direct DNA-damaging potential of the hepatocarcinogen DEN. In contrast, a statistically significant ($P < 0.001$) decrease in the total single-stranded DNA generation was observed in the BC-treated group 24 h after DEN injection (Figure 3). Moreover, the native double-stranded DNA in group C animals was almost two-fold higher than in group B animals (Figure 3). Table 3 shows the number of single-strand breaks/DNA 24 h after DEN injection in the presence or absence of BC supplementation. There was a significant ($P < 0.01$) increase in the number after DEN treatment alone compared with control counterparts. On the other hand, hepatic DNA in the BC-supplemented DEN group showed 59.66% fewer single-strand breaks/DNA compared with DEN controls (group B). Supplementation of the basal diet with BC alone for 15 days (group D) did not have any DNA-damaging effect, as revealed by the insignificant difference in the generation of single-strand breaks/DNA compared with normal controls, i.e. group A (Table 3).

### DISCUSSION

The participation of CAs in tumour initiation and promotion is well documented and is suggested by the association of specific chromosomal rearrangements with particular cancers (Gilbert, 1983).

CAs are known to be important somatic mutations and are clearly involved in the origin as well as progression and diversification of certain types of cancers (Nowell, 1976; Land et al, 1983), but it is difficult to characterize aberrations crucial for initiation and early stages of tumour development. However, in view of the acknowledged importance of CAs as a hallmark of stage of progression in multistage carcinogenesis, we attempted to gain an insight into the critical CAs involved in the early stages of hepatic neoplasia accompanied by DNA chain breaks induced by the potent hepatocarcinogen DEN and its possible alterations by BC – a well-established anti-tumour micronutrient.

Induction of pro-oxidant status causes lipid peroxidation because the polyunsaturated fatty acid side chains of membrane lipids are particularly sensitive to oxidation. Lipid hydroperoxides and their degradation products may act as clastogenic factors (low molecular weight components that break chromosomes at the same or remote tissues). These lipid hydroperoxides and active oxygen species mostly act as secondary agents that produce secondary DNA damage in reactions with cellular molecules other than DNA and are thus potent inducers of CAs (Cerutti, 1985).

Further, a study of the early stages of hepatocarcinogenesis became possible with the use of cell lines that were derived from rat liver after the exposure of rats to a carcinogen for a limited time period (Kerler and Rabes, 1988). A minimal alteration in chromosomal pattern was found to correlate with an earlier preneoplastic stage, as evidenced by chromosomal analysis of DEN-induced tumorigenic and non-tumorigenic rat liver cell lines (Holecek et al, 1989). In our present study, a variable increase in CAs in rat liver cells at various time intervals was observed during the early preneoplastic stage of DEN-induced hepatocarcinogenesis. Our results with respect to CAs are in agreement with those previously observed during preneoplasia in rat liver cells using the same hepatocarcinogen (Grover and Fisher, 1971; Hitachi et al, 1974).

Another striking observation of the present study was the BC-mediated suppression of structural as well as numerical aberrations. It has been well established that in the majority of malignant tumours, the neoplastic cells have undergone chromosomal alterations that are viable in extent but are often highly complex, usually indicating structural and numerical aberrations. Again, a high rate of chromosome breakage, which represents structural aberrations, has been aetiologically associated with the initiation of the carcinogenic process. Thus, if the breakage lesions are found to be non-random and if the breakage loci happen to be those that are virtually linked to tumorigenesis, then the probability of tumorigenesis would increase drastically in the target tissue (Dave et al, 1994). In the light of above, the role of BC in suppressing the structural and numerical aberrations as observed here may reflect the ability of BC to counteract the initiation of rat liver carcinogenesis. This finding corroborates our earlier observation that the anti-carcinogenic potential of BC is maximally observed during the initiation phase of DEN-induced hepatocarcinogenesis in rats (Sarkar et al, 1995*a*).

Carcinogen-induced DNA damage, DNA repair and sister chromatid exchange (SCE) are significant events during the initiation stage of carcinogenesis (Popescu et al, 1984). Very little information is available regarding BC inhibition of experimental carcinogenesis at the chromosomal level. In one study, Manoharan and Banerjee (1985) reported that in the mammary epithelial cell transformation model in organ culture, the presence of BC during 24 h treatment (initiation stage) of the glands with the carcinogens, e.g. DEN, dimethylbenz(*a*)anthracene and methyl nitrosourea, caused a highly significant reduction of SCE induced by the same carcinogens.

At present the intricate mechanism of this anticlastogenic response of BC is unknown. It is well known that DNA strand breaks responsible for chromosomal alterations are generated from DNA-base lesions induced by most chemical mutagens. These DNA-base lesions are generally repaired by the excision–repair system (Friedberg et al, 1979). One hypothesis is that the in vivo anticlastogenic effect of BC, as observed here, may be due to the promotion of excision–repair activity. The substantial decrement of the single-strand breaks can explain one possible mechanism of the anticlastogenic potential of BC. Again, it has also been established that DNA double-strand breaks (DDBs) are generated from mutagen-induced DNA lesions in the S-phase of cell cycle. It is considered that DDBs are repaired by post-replicational repair in the $G_2$ phase and that unrepaired DDBs result in breakage-type CAs (Kihlman et al, 1982). In this context, the suppression of breakage-type aberrations by BC may be due to a modification of the capability of the post-replicational repair of DDBs.

Regardless of the mechanism, the results of this study provide strong evidence that the antioxidant BC triggers a unique protective effect against the induction of CAs and DNA chain breaks by a potent hepatocarcinogen, DEN. The biological and molecular response of the dietary micronutrient BC observed here may have value as a chemopreventive agent in the war on human cancer.

### ACKNOWLEDGEMENT

Alok Sarkar, Anupam Bishayee and Jayasri Basak are indebted to the council of Scientific and Industrial Research, Government of India, for the award of a Senior Research Fellowship [9/96 (225)/94-EMR-I], Research Associateship [9/96 (262)/95-EMR-I] and Pool Officership [13(6580-A)/93-Pool] respectively while Ranjan Basak is grateful to DST for financial support during the execution of the study.

### REFERENCES

Ahnstrom G and Erixon K (1973) Radiation induced strand breakage in DNA from mammalian cells. Strand separation in alkali solution. *Int J Radiat Biol Relat Stud Phys Chem Med* **23**: 285–289

Basak J (1996) Estimation of single-strand breaks induced in the dried film of DNA by high energy alpha particle from a cyclotron. *Ind J Biochem Biophys* **33**: 35–38

Birnboim HC and Jevcak JJ (1981) Fluorimetric method for rapid detection of DNA strand breaks in human white blood cells produced by low doses of radiations. *Cancer Res* **41**: 1889–1892

Bishayee A and Chatterjee M (1995) Inhibition of altered liver cell foci and persistent nodule growth by vanadium during diethylnitrosamine-induced hepatocarcinogenesis in rats. *Anticancer Res* **15**: 455–462

Bradley MO, Erickson LC and Kohn KW (1978) Non-enzymatic DNA strand breaks induced in mammalian cells by fluorescent light. *Biochim Biophys Acta* **520**: 11–20

Cerutti PA (1985) Prooxidant states and tumor promotion. *Science* **227**: 375–381

Dave BJ, Hsu TC, Hong WK and Pathak S (1994) Nonrandom distribution of mutagen-induced chromosome breaks in lymphocytes of patients with different malignancies. *Int J Oncol* **5**: 733–740

Erickson LC, Osieka R, Sharkey NA and Kohn KW (1980) Measurement of DNA damage in unlabelled mammalian cells analyzed by alkaline elution and a fluorimetric DNA assay. *Anal Biochem* **106**: 169–174

Friedberg EC, Ehmann UK and Williams JI (1979) Human diseases associated with defective DNA repair. *Adv Radiat Biol* **8**: 85–174

Gerster H (1993) Anticarcinogenic effect of common carotenoids. *Int J Vit Nutr Res* **63**: 93–121

Gerster H (1995) β-Carotene, vitamin E and vitamin C in different stages of experimental carcinogenesis. *Eur J Clin Nutr* **49**: 155–168

Gerster H (1996) Intermediate cancer biomarkers and their use in beta-carotene studies in humans. *Int J Vit Nutr Res* **66**: 3–18

Gilbert F (1983) Chromosomes, genes and cancer: a classification of chromosomal abnormalities in cancer. *J Natl Cancer Inst* **71**: 1107–1114

Grover S and Fisher P (1971) Cytogenetic studies in Sprague-Dawley rats during the administration of a carcinogenic nitroso compound – diethylnitrosamine. *Eur J Cancer* **7**: 77–82

Gupta RC (1984) Nonrandom binding of the carcinogen N-hydroxy-2-acetylaminofluorene to repetitive sequences of rat liver DNA in vivo. *Proc Natl Acad Sci USA* **81**: 6943–6947

Gutin PH, Hilton J, Fein VJ, Alien AE and Walder MD (1977) SI nuclease from *Aspergillus oryzae* for the detection of DNA damage and repair in the gamma irradiated intracerebral rat gliocercoma IL. *Radiat Res* **72**: 100–106

Hitachi PM, Yamada K and Takayama S (1974) Diethylnitrosamine-induced chromosomal changes in rat liver cells. *J Natl Cancer Inst* **53**: 507–516

© Cancer Research Campaign 1997

Holecek BU, Kerler RR and Rabes HM (1989) Chromosomal analysis of a diethylnitrosamine-induced tumorigenic and nontumorigenic rat liver cell line. *Cancer Res* **49**: 3024–3028

Horiuchi T, Ito K, Suzuki M and Umeda M (1984) Sensitive induction of chromosome aberrations in the in vivo liver cells of rats by N-nitrosodiethylamine. *Mutation Res* **140**: 181–185

Kanter PM and Schwartz HW (1979) Hydroxyapatite batch assay for quantitation of cellular DNA damage. *Anal Biochem*, **97**: 77–84

Kerler R and Rabes HM (1988) Preneoplastic rat liver cells in vitro: slow progression without promoters, hormones or growth factors. *J Cancer Res Clin Oncol* **114**: 113–123

Kihlman BA, Hansson K and Andersson HC (1982) The effects of post-treatments with caffeine during S and $G_2$ on the frequencies of chromosomal aberrations induced by thiotepa in root tips of *Vicia fava*. *Environ Exp Bot* **20**: 271–286

Kohn KW and Ewig RAG (1973) Alkaline elution analysis approach to the study of DNA single strand interruptions in cells. *Cancer Res* **33**: 1849–1853

Kohn KW, Erickson LC, Ewig AG and Friedman CA (1976) Fractionations of DNA from mammalian cells by alkaline elusion. *Biochemistry* **45**: 4629–4637

Krinsky NJ (1993*a*) Micronutrients and their influence on mutagenicity and malignant transformation. *Ann NY Acad Sci* **686**: 229–242

Krinsky NJ (1993*b*) Actions of carotenoids in biological systems. *Annu Rev Nutr* **13**: 561–587

Land H, Parada LF and Weinberg RA (1983) Cellular oncogenes and multistep carcinogenesis. *Science* **222**: 771–778

Manoharan K and Banerjee MR (1985) β-carotene reduces sister chromatid exchanges induced by chemical carcinogens in mouse mammary cells in organ culture. *Cell Biol Int Rep* **9**: 783–789

Moreno FS, Rizzi MBSL, Dagli MLZ and Penteado MVC (1991) Inhibitory effect of β-carotene on preneoplastic lesions induced in Wister rats by the resistant hepatocyte model. *Carcinogenesis* **12**: 1817–1822

Morgan AR and Pullyblank DE (1974) Native and denatured DNA crosslinked and palindromic DNA analyzed by a sensitive fluorimetric procedure. *Biochem Biophys Res Commun* **61**: 396–403

Nowell PC (1976) The clonal evolution of tumor cell populations. *Science* **194**: 23–28

Paterson MC (1978) Use of purified lesion-recognizing enzymes to monitor DNA repair in vivo. *Adv Radiat Biol* **7**: 1–53

Popescu NC, Ansbaugh SC and Dipaolo JA (1984) Correlation of morphological transformation to sister chromatid exchanges induced by split doses of chemical or physical carcinogens on cultured Syrian hamster cells. *Cancer Res* **44**: 1933–1938

Randerath K, Reddy MV and Gupta RC (1981) $^{32}$P-labelling test for DNA damage. *Proc Natl Acad Sci USA* **78**: 6126–6129

Reddy MV, Gupta RC, Randerth E and Randerath K (1984) $^{32}$P-postlabelling test for covalent DNA binding of chemicals in vivo: application to a variety of aromatic carcinogens and methylating agents. *Carcinogenesis* **5**: 231–243

Rousseau EJ, Davison AJ and Dunn B (1992) Protection by β-carotene and related compounds against oxygen-mediated cytotoxicity and genotoxicity: implications for carcinogenesis and anticarcinogenesis. *Free Rad Biol Med* **13**: 407–433

Rydberg B (1975) The rate of strand separation in alkali of DNA of irradiated mammalian cells. *Radiat Res* **61**: 274–287

Rydberg B (1980) Detection of induced DNA strand breaks with improved sensitivity in human cells. *Radiat Res* **81**: 492–495

Sarkar A, Mukherjee B and Chatterjee M (1994*a*) Inhibitory effect of β-carotene on chronic 2-acetylaminofluorene-induced hepatocarcinogenesis in rat: reflection in hepatic drug metabolism. *Carcinogenesis* **15**: 1055–1060

Sarkar A, Mukherjee B, Rana MP and Chatterjee M (1994*b*) Comparative patterns of hepatic drug metabolizing enzymes and their possible correlation with chromosomal aberrations in transplantable murine lymphoma: a time course study. *Cancer Invest* **12**: 477–483

Sarkar A, Bishayee A and Chatterjee M (1995*a*) Beta-carotene inhibits lipid peroxidation and red blood cell membrane protein damage in experimental hepatocarcinogenesis. *Cancer Biochem Biophys* **15**: 111–125

Sarkar A, Mukherjee B and Chatterjee M (1995*b*) Inhibition of 3′-methyl-4-dimethylaminoazobenzene-induced hepatocarcinogenesis in rat by dietary β-carotene: changes in hepatic anti-oxidant defence enzyme levels. *Int J Cancer* **61**: 799–805

Sheriden RB, III and Huang PC (1977) Single strand breakage and repair of eukaryotic DNA as assayed by SI nuclease. *Nucleic Acids Res* **4**: 299–318

Tsuda H, Uehara N, Iwahori Y, Asamoto M, Iigo M, Nagao M, Matsumoto K, Ito M and Hirono I (1994) Chemopreventive effects of beta-carotene, alpha-tocopherol and five naturally occurring antioxidants on initiation of hepatocarcinogenesis by 2-amino-3-methylimidazo [4, 5-f] quinoline in the rat. *Jpn J Cancer Res* **85**: 1214–1219

Van Poppel G (1993) Carotenoids and cancer: an update with emphasis on human intervention studies. *Eur J Cancer* **29**: 1335–1344

© Cancer Research Campaign 1997